# Expression of muscarinic receptors in human and mouse sclera and their role in the regulation of scleral fibroblasts proliferation

**Published:** 2009-06-30

**Authors:** V. A. Barathi, S. R. Weon, R. W. Beuerman

**Affiliations:** 1 Singapore Eye Research Institute, Singapore; 2 Department of Ophthalmology, Yong Loo Lin School of Medicine, National University of Singapore, National University Hospital, Singapore

## Abstract

**Purpose:**

To determine the expression of muscarinic receptor subtypes (mAChRs) in human and mouse scleral fibroblasts (SFs), to investigate the mechanism that mediate the role mAChRs play in cell proliferation, and to explore the underlying intracellular signaling pathways involved in mouse SFs with treatment of muscarinic agents.

**Methods:**

Reverse transcription polymerase chain reaction (RT-PCR) was used to detect mRNA expression of mAChRs in the human and mouse sclera. Western blot analysis and immunocytochemistry were used to detect proteins of mAChRs in the cultured SFs. An immunohistochemical study was used to further detect the presence of mAChR proteins in frozen scleral sections. BrdU (5-bromo-2-deoxyuridine ) cell proliferation assay was performed to measure DNA synthesis. Enzyme linked immunosorbent assay (ELISA) was used to measure in vitro kinase activity for epidermal growth factor receptor (EGF-R), fibroblast growth factor (FGF-2), transforming growth factor (TGF)-β1, and extracellular signal-regulated kinase (ERK)1/2. Expressions of epidermal growth factor-receptor (EGF-R); protein kinase C (PKC); Proline-rich tyrosine kinase 2 (Pyk-2), v-raf murine sarcoma viral oncogene homolog B1 (B-Raf), Rat Sarcoma (Ras), c-Jun N-terminal kinases (JNK1/2), and ERK1/2 were detected by immunoblot.

**Results:**

mAChR for subtypes M_1_-M_5_ were detected in both mouse and human SFs by protein, cellular, and mRNA analysis. EGF-R, PKC, Pyk-2, B-Raf, Ras, JNK1/2, and ERK1/2 were activated after treatment by agonists and antagonists, indicated by changes in phosphorylation of these proteins. Atropine abolished the carbachol-induced activation of SF cell proliferation in a concentration-dependent manner. Carbachol also activated p42/44  mitogen-activated protein kinase (MAPK) and Ras in a time-dependent manner. Muscarinic agents also modulated fibroblast growth factor expression in these cells.

**Conclusions:**

This study confirms the presence and functional role of all five mAChRs in human and mouse SFs. These results show that proliferative responses of SFs to muscarinic receptor stimulation are mediated via the activation of the classical MEK-ERK-MAPK cascade.

## Introduction

Myopia is a common problem in Asia [1,2], and the prevalence of myopia is increasing worldwide. It is a socioeconomic problem, and high myopia, which is sight threatening, is becoming more common [3]. In Taiwan, myopia is considered a leading cause of blindness due to the number of people with high myopia. Therefore, preventing the progression of myopia is an active area of investigation. Atropine, a pan muscarinic antagonist [4,5], and pirenzepine, an antagonist more specific for M_1_ [6], have been found effective in clinical trials with children in preventing myopia progression. These two drugs have also been tested in studies using animal models of myopia [7,8] and were found to block axial elongation during the development of form-deprivation myopia. Cellular signals acting on the main cell type of the sclera, the fibroblast, may direct the growth process resulting in myopia. As muscarinic antagonists inhibit scleral growth in children, the focus has been on muscarinic receptors.

By understanding the specific pharmacological and molecular mechanisms of the action of muscarinic antagonists on the individual muscarinic receptors, insights into the molecular signaling pathway in axial elongation may be developed [9]. Another outcome of this approach is the development of specific blockers overcoming some of the side effect issues associated with atropine, a pan muscarinic antagonist. Recently, we have developed a mouse model of experimental myopia, and we demonstrated that the observed axial elongation was due to growth of the posterior chamber of the eye [10].

Many studies have reported that the muscarinic receptors have important roles in the nervous system [11]. However, recent studies have suggested that muscarinic receptors are widely expressed in non-neuronal cells such as muscle fibers and epithelial, endothelial, and immune cells [12,13]. Muscarinic acetylcholine receptors are widely distributed within the eye [14], once again making the identification of the site of action difficult. Muscarinic toxins from green mamba venom modulate the proliferative actions of mAChRs in mouse and human scleral fibroblasts [15]. The site of action of the muscarinic cholinergic antagonists in human myopia is not well known, although effects on the retina [16] and the sclera [17] have been considered.

Since mAChRs are known to transactivate growth factor receptors [18], the action of muscarinic antagonists may also be mediated indirectly through receptor tyrosine kinases, which could then be distributed throughout signaling pathways within the sclera fibroblast. Tyrosine kinases are important components of signaling pathways that couple cell surface receptors to the regulation of cellular activities such as gene expression, proliferation, and ion channel modulation. Studies show that growth factors, cytokines, integrins, antigens, and G protein coupled receptors (GPCRs) also utilize tyrosine kinases to transduce intracellular signals [19-22]. In fact, GPCRs are the most frequent targets of pharmacological therapies.

A recent study has demonstrated that multiple mAChRs occur in mammals including humans, and the distribution of these receptors is tissue specific [17]. In human ocular tissues, the M_3 _receptor is the main mAChR in the cornea, iris, ciliary body, and epithelium of the crystalline lens [23,24]. The M_3_ and M_4_ receptors are the main mAChRs in the retina [16], M_1_-M_4_ are the main ones in lung fibroblasts [25], and M_1_, M_3_, and M_5_ are functional in human labial salivary glands [26]. The biology of the subtypes of mAChRs have not been explored in detail in the eye, but at both mRNA and protein levels, all five mAChRs were detected in the human sclera [27], tree shrew sclera [28], guinea pig sclera [29], mouse sclera [30], monkey’s eyelid conjunctival epithelial cells, meibomian glands and supra-basal layers of the skin [31]. However, the functional significance of cholinergic receptors in scleral fibroblasts (SFs) remains to be studied in detail.

Thus, we suggest that mAChRs regulation are necessary for the growth of the sclera in experimental myopia. It is shown here that mAChRs mediate the proliferation of human and mouse SFs, which in turn may have a role in scleral remodeling. The aim of our study was to determine the expression of mAChRs in human and mouse SFs and to investigate whether mAChRs can mediate SF cell proliferation.

## Methods

### Human sclera

Human scleral tissues (n=19) harvested within 24 h from cadaver eyes were provided by the Singapore Eye Bank. The protocol was approved by the Institutional Review Board of the Singapore Eye Research Institute and complied with the tenets of the Declaration of Helsinki.

### Animals

BALB/c mice (n=102) were obtained from the animal holding unit of the National University of Singapore. All animals were housed in standard mouse cages at 25 °C on a schedule of 12 h:12 h of light on and off with mouse pellet and water available ad libitum, and the water was regularly changed. Approval was obtained from the SingHealth IACUC, and all procedures performed in this study were in accordance with the Guide for the Care and Use of Laboratory Animals and ARVO recommendations for animal experimentation.

### Culture of human and mouse scleral fibroblast cells

Scleral rims from donor human cadaver eyes (n=16) and eight-week-old mouse sclera (n=150, 10 sclera/batch) from post-mortem eyes were obtained. The whole sclera was dissected very carefully and washed with cold phosphate buffered saline (PBS) three times. The human scleral tissue was cut into 2 mm×2 mm pieces, and the mouse scleral tissue was cut into 1 mm×1 mm pieces. Scleral tissues were dispersed by incubating with 0.25% trypsin/0.5 mM EDTA (Sigma, Hamburg, Germany) for 5 min at 37 °C. The tissues were placed on 60 mm×15 mm cell culture dishes (Greiner, Dresden, Germany) with a thin layer of Dulbecco’s modified Eagle’s medium (DMEM; Sigma) supplemented with 10% fetal bovine serum (FBS; Gibco BRL, San Francisco, CA), 50 U/ml penicillin, and 50 µg/ml streptomycin and amphotericin B (Sigma). Cells were grown in a humidified atmosphere of 5% CO_2_ at 37 °C. After initial cell outgrowth from the explants was observed, the volume of the medium was increased. The cultures were passaged on reaching 80% confluence. All cells used in experiments were between passages 1 and 2.

### Cell proliferation assay

SFs were passaged from human and mouse scleral tissues and used between passages 1 and 2. Cell proliferation was assessed by measuring 5-bromo-2-deoxyuridine (BrdU) incorporation during DNA synthesis in proliferating cells (Oncogene, Cambridge, MA). For the cell proliferation assay, 100 μl of passaged SFs (1×10^5^ cells/ml) were seeded into 96 well plates containing DMEM with 10% FBS. Cells were allowed to attach to the substratum for 24 h. The following day, the medium was replaced with fresh DMEM containing 10 µM BrdU (Oncogene) to be incorporated into newly synthesized DNA. Cells were then treated with experimental concentrations of the pan muscarinic receptor agonist, carbachol (Sigma); muscarinic toxin-1 (MT-1, a well characterized M_1_-specific agonist derived from snake venom; Peptides International Inc., Louisville, KY); or the pan receptor antagonist, atropine (Sigma); or M_1_ selective antagonist, pirenzepine (Sigma); muscarinic toxin-7 (MT-7, M_1_ specific antagonist derived from snake venom; Peptides International Inc.); M_2_/M_4_ receptor antagonist, himbacine; or the M_3_ receptor antagonist, 4-(diphenylacetoxy-N-methyl piperidine) methiodide (4-DAMP). To study the combined effect of atropine and carbachol, SFs were pre-treated with atropine (0.1–100 µM) 3 h before being incubated with equimolar carbachol.

After 24 h of incubation, the culture medium was removed, cells were fixed and permeabilized, and the DNA was denatured to enable antibody binding to the incorporated BrdU. Anti-BrdU antibody was added to the wells and incubated for 1 h at room temperature. Unbound antibodies were washed away, and horseradish peroxidase-conjugated goat-anti mouse was added. A substrate solution was added to each well, resulting in a color change proportional to the amount of DNA synthesis in the cells. The color reaction was stopped, and the optical density was determined using a Spectrafluor Plus microplate reader (TECAN, Durham, NC), set to 450 nm-595 nm.

### Immunohistochemistry and immunocytochemistry

The whole mouse eye (two months old, n=3) and human sclera (n=3) was embedded in Optimal Cutting Temperature (OCT) compound at −20 °C for 1 h. Prepared tissue blocks were sectioned with a cryostat at 5 microns and collected on clean polysine™ glass slides (Thermo Fisher Scientific, Newington, CT). Sections were air dried at room temperature (RT) for 1 h and fixed with 4% paraformaldehyde for 10 min. After washing three times for 5 min each with 1X PBS and then adding 4% goat serum diluted with 1X PBS as a blocking buffer, slides were covered and incubated for 1 h at RT in a humidified chamber. After rinsing with 1X PBS, a specific primary antibody for M_1_-M_5_ (Biogenesis, Poole, England) was diluted with 2% goat serum (1:100) and incubated overnight at 4 °C in a humid chamber. After washing three times for 10 min each with 1X PBS, fluorescein-labeled goat anti-rabbit secondary antibody (1:200; Sigma) was applied and incubated for 90 min at RT. After washing and air drying, slides were mounted with an antifade medium containing DAPI (4, 6-diamidino-2-phenylindole; Vectashield; Vector Laboratories, Burlingame, CA) to visualize nuclei. For negative controls, primary antibodies were omitted.

In parallel, mouse and human SFs were cultured on sterile chamber slides. Cells were washed with PBS and fixed with ice-cold methanol:acetone (1:1) at −20 °C for 10 min and air-dried. Cells were permeabilized with 0.5% Triton X-100 in PBS for 2 min at RT. Non-specific sites were blocked with 1% bovine serum albumin (BSA), 0.3% Triton X-100, and PBS for 30 min at RT for the cells. The cells were then processed and stained as described above. The cells were mounted on coverslips with Flurosave mounting medium (Calbiochem, San Diego, CA). A fluorescence microscope (Axioplan 2; Carl Zeiss Meditec GmbH, Oberkochen, Germany) was used to examine the slides and images captured. Experiments were repeated in triplicate from three different samples.

### RNA isolation and RT–PCR

The mouse globe (n=50, 10 sclera/batch) was washed with a buffered saline solution to remove traces of blood and other material. The sclera behind the optic nerve head was dissected from each eye. The whole retina including the retinal pigment epithelium (RPE) was stripped off from the sclera, and choroid was removed with a No. 10 scalpel blade. The whole sclera was then immediately frozen in liquid nitrogen.

Total RNA was extracted from human and mouse scleral tissues (TRIzol reagent; Invitrogen-Gibco, Carlsbad, CA). Genomic DNA was removed by digestion with DNase I (Amp Grade; Invitrogen-Gibco) for 15 min at RT. One microgram of total RNA was reverse-transcribed with random hexamers by using a first-strand cDNA synthesis kit (Invitrogen-Gibco). To standardize and evaluate scleral gene expression, aliquots of the same cDNA preparation were used as templates in all polymerase chain reactions (PCR) reactions. PCR amplification (PTC-200 Peltier Thermal Cycler; MJ Research, Ramsey, MN) was performed using Taq DNA polymerase (Promega, Madison, WI). Each reaction confirmed that the PCR products were not saturated. The primers for muscarinic receptors were constructed with the Primer Express Software version 1.0 (PE Applied Biosystems, Foster City, CA) using a mouse and human specific acetylcholine muscarinic receptor subtypes sequence (Table 1 and Table 2, respectively). Total RNA extracted from the mouse brain was used as a positive control. PCR amplification of *β-actin* was performed in parallel as an internal control to detect genomic DNA contamination. The amplified products were analyzed by electrophoresis on 1.2% and 2.5% agarose-TAE gels (human and mouse sclera, respectively) and photographed under ultraviolet (UV) light. All experiments were done in triplicate.

**Table 1:** Primer sequences and sizes of mouse PCR products.

**Gene**	**Primers**	**Size**
*M_1_*	F: 5′-TCCCTCACATCCTCCGAAGGTG-3′	139 bp
	R: 5′-CTTTCTTGGTGGGCCTCTTGACTG-3′	
*M_2_*	F: 5′-CTGGAGCACAACAAGATCCAGAAT-3′	69 bp
	R: 5′-CCCCCTGAACGCAGTTTTCAGT - 3′	
*M_3_*	F: 5′-GCAAGACCTCTGACACCAACT-3′	91 bp
	R: 5′-AGCAAACCTCTTAGCCAGCG-3′	
*M_4_*	F: 5′-CGGCTACTGGCTCTGCTACGTCAA-3′	122 bp
	R: 5′-CTGTGCCGATGTTCCGATACTGG-3′	
*M_5_*	F: 5′-TAGCATGGCTGGTCTCCTTCA-3′	76 bp
	R: 5′-CGCTTCCCGACCAAGTACTG-3′	

**Table 2:** Primer sequences and sizes of human PCR products.

**Gene**	**Primers**	**Size**
*M_1_*	F: 5′-CAGGCAACCTGCTGGTACTC-3′	538 bp
	R: 5′-CAGGCAACCTGCTGGTACTC-3′	
*M_2_*	F: 5′-CTCCTCTAACAATAGCCTGG-3′	654 bp
	R: 5′-GGCTCCTTCTTGTCCTTCTT-3′	
*M_3_*	F: 5′-GGACAGAGGCAGAGACAGAA-3′	560 bp
	R: 5′-GAAGGACAGAGGTAGAGTGG-3′	
*M_4_*	F: 5′-ATCGCTATGAGACGGTGGAA-3′	503 bp
	R: 5′-GTTGGACAGGAACTGGATGA-3′	
*M_5_*	F: 5′-ACCACAATGCAACCACCGTC-3′	752 bp
	R: 5′-ACAGCGCAAGCAGGATCTGA-3′	
*β-actin*	F: 5′-CACTCTTCCAGCCTTCCTTC-3′	314 bp
	R: 5′-CTCGTCATACTCCTGCTTGC-3′	

The *β-actin* PCR primer pair was selected to span a 206 bp intron [61].

### Relative and absolute quantitative real-time PCR

Real-time PCR primers for transcripts of *M_1_*-*M_5_* were purchased from Applied Biosystems Inc. (Taqman Gene Expression System; ABI, Foster City, CA), and Quantum RNA classic II 18S Internal Standard (Ambion, Austin, TX) was used as an endogenous control. The 18S primer-competimer ratio of 1:4 was used in all experiments. Real-time PCR reactions were performed on a sequence detection system (Prism 7700; ABI) with 500 ng of total cDNA per reaction in a final volume of 25 µl. The C_T_ (threshold cycle) of each reaction was obtained by using a constant threshold with the  18S rRNA used as an internal control. ∆C_T_ was calculated by subtracting the average C_T_ of 18S rRNA from the average C_T_ of the target gene. All experiments were performed in triplicate. This was repeated with five different batches of samples. The conditions for the PCR were as follows: 55 °C for 2 min, 95 °C for 10 min and 40 cycles each of 95 °C for 30 s, 60 °C for 30 s, and 72 °C for 2 min. The comparative quantification values were obtained from the threshold cycle (C_T_) number at which the increase in signal was associated with an exponential growth of PCR products and this was detected in SDS (Satellite Data System) software in ABI PRISM® 7700 Sequence Detection System. The PCRs were performed (n=5) for each sample-primer set, and the mean of the experiments was used as the relative quantification value. For all *M_1_*-*M_5_* probes, the ABI-confirmed amplification specificity of TaqMan probes and the reaction products were separated on 3% agarose gel, stained with ethidium bromide for visual confirmation of PCR products, and sequenced.

### Data analysis by comparative C_T_ method

The C_T_ value represented the PCR cycle at which an increase in reporter fluorescence (**∆**Rn) above the line of the optimal value (optimal **∆**Rn) was first detected. The calculation for the comparative C_T_ (∆∆C_T_) method was previously described [32]. 18S rRNA was used as an endogenous internal control. The average C_T_ value of all muscarinic receptor genes and 18S rRNA was calculated from each experiment of a triplicate, and this was used for all five samples. Finally, the mean C_T_ value was calculated from the five average C_T_ values. The **∆**C_T_ value was determined by subtracting the corresponding mean 18S rRNA C_T_ value from the mean of the *M_1_*-*M_5_* C_T_ value. The standard deviation of the difference was calculated from the standard deviations of the gene of interest and the corresponding 18S rRNA values. The **∆∆**C_T_ value was obtained by subtracting the **∆**C_T_ calibrator value. This was the subtraction of an arbitrary constant so the standard deviation of the **∆∆**C_T_ was similar to the standard deviation of the **∆**C_T_ value. The expression level of each gene in the mouse brain was used for calibration. **∆∆**C_T_ of the scleral sample was calculated by subtracting the **∆**C_T_ of the mouse brain from **∆**C_T_ of the scleral sample. The relative change of scleral samples compared with brain samples was determined as 2^-^**^∆∆^**^CT^. Statistical analysis was performed by ANOVA. The expression levels of the genes for M_1_–M_5_ in the sclera were compared by the Fisher LSD (Least Significant Difference) test. A probability level of p<0.05 was considered statistically significant.

### Phospho-p42/44 MAPK activation assay

Mouse SF cell lysate was exposed to atropine and carbachol at 0, 0.1, 1, 10, and 100 µM for 0.5 h. Phospho-p42/44 mitogen-activated protein kinase (MAPK) assay kit were purchased from Assay Design (Ann Arbor, MI), and all procedures were conducted according to the instructions provided by the company. A monoclonal antibody specific for phospho-p42/44 MAPK was pre-coated onto a 96 well microtiter plate. Standards and samples were added into the wells, and p42/44 MAPK became bound to the immobilized antibody. Proteins were removed by washing, and an enzyme-linked monoclonal antibody specific for p42/44 MAPK was added to the wells. Washed again to remove any unbound antibody-enzyme reagent and the calorimetric, a substrate solution was added to the wells. Color was developed in proportion to the amount of p42/44 MAPK bound in the initial step. The intensity of the color was measured at 450 nm with an enzyme-linked immunosorbent assay (ELISA) reader (TECAN, Durham, NC) to determine the levels of phosphorylated p42/44 MAPK proteins.

### Western blotting-protein expression of mAChRs

The nearly confluent mouse and human SFs were homogenized in ice-cold radio immunoprecipitation assay (RIPA) buffer containing proteinase inhibitors (10 mM Tris-HCl [pH 7.4], containing 150 mM NaCl, 1% deoxycholic acid, 1% Triton X-100, 0.1% SDS, 1 mM EDTA, 10 mg/ml phenylmethylsulfonyl fluoride, 5 U/ml aprotinin, and 100 nM sodium orthovanadate) and phosphatase inhibitors (Pierce, Rockford, IL). After homogenization, the samples were centrifuged at 14,000x g for 10 min at 4 °C, and the supernatants were used as total cell lysates.

Total protein concentration was determined using a direct colorimetric (DC) protein assay reagent (Bio-Rad, Hercules, CA). Theses proteins were used to detect the muscarinic receptors in both human and mouse SF using immunoblot. Proteins in the supernatant were separated by SDS–PAGE, transferred to nitrocellulose membranes, blocked in 5% BSA in TBST (10 mM Tris-HCl [pH 8.0], 150 mM NaCl, and 0.05% Tween-20) for 2 h at RT, and incubated with the same anti-muscarinic receptor antibodies described earlier at a dilution of 1:1000 to detect the mAChRs proteins.

### Analysis of Erk1/2 and Ras phosphorylation by carbachol and FGF-2 on mouse SF

The mouse SF cells were cultured in a serum-containing medium in six wells (2×10^5 ^cells/well). Cultured mouse SFs were starved for 24 hours and then was incubated with 50 mg/ml carbachol or 50 ng/ml FGF-2 for 0.5, 1, 2, 6 h and over night to detect the optimal time required for MAPK (ERK1/2) and Ras phosphorylation in these cells. For detection, western blot was performed as described below.

### Analysis of phosphorylation by muscarinic agents in mouse SF

The mouse SF cells were cultured in a serum-containing medium in six wells (2×10^5^ cells/well). From the earlier analysis, we found that 30 min was the optimal time to detect the ERK1/2 phosphorylation. Hence, we have treated the mouse SFs with freshly prepared atropine, pirenzepine, and carbachol at a concentration of 50 µM and MT-1 and MT-7 at a concentration of 0.5 µM for 30 min to determine the signaling phosphorylation of EGF-R, PKC, Pyk2, B-Raf, Ras, JNK1/2 and ERK1/2 proteins with the effect of muscarinic agents. Protein extraction and electrophoresis were performed as described above.

For detection, the membrane was incubated with anti-Ras clone RAS10, phospho-EGF-R, PKC, Pyk2, B-Raf, Ras, JNK1/2, and ERK1/2 antibodies at the dilution of 1:1000 according to the instructions provided by the company (Millipore, Billerica, MA) for 2 h at RT, and β-tubulin was used as a loading control and was incubated for 1 h at RT.

All three experiments’ membranes were washed three times in TBST and incubated with HRP-conjugated secondary antibody (Chemicon International, Temecula, CA) at a dilution of 1:2500 for 1 h at RT. Immunoreactive bands were visualized using the enhanced chemiluminescence method (GE Healthcare, Buckinghamshire, UK). The membrane was wrapped in plastic and placed against X-ray film, exposed for an appropriate length of time (30 s to 5 min), and developed (Kodak, Rockford, IL).

### Growth factor receptors ELISA

Mouse SF cell lysate was exposed to atropine and carbachol at concentrations of 0, 0.1, 1, 10, and 100 µM for 24 h. Cellular EGF, fibroblast growth factor-2 (FGF-2), and transforming growth factor (TGF)-β1 receptors in the presence of muscarinic agents was quantified by an ELISA. Equal amounts of samples or standards were added to each well (anti-EGF-R, -FGF-2, and -TGF-β1 antibody pre-coated) and incubated for 2 h at RT. The wells were aspirated and washed with PBST. Anti-EGF, -FGF-2 (Chemicon International Inc.), and -TGF-β1 (Amersham Pharmacia Biotech, Buckinghamshire, UK) mouse IgG conjugated with horse-radish peroxidase was added to the respective wells and incubated for 2 h at RT. Substrate solution was added to each well, resulting in a color change proportional to the amount of EGF-R, FGF-2, and TGF-β1 present in the samples. Samples were allowed to develop color for 20 min at RT. Adding stop solution stopped the reaction, and the optical density was determined within 30 min using Spectrafluor Plus microplate reader (TECAN) at dual wavelengths of 450 nm and 620 nm. Quantification was achieved by the construction of a standard curve using the known concentration of EGF, FGF-2, and TGF-β1 receptors.

### Data analysis

Statistical comparisons between experimental groups were conducted using Student’s *t*-test or one-way ANOVA (Statistica 6.0; SPSS, Chicago, IL) followed by Tukey post hoc test. A significance level of p<0.05 was used. Data are presented as means±SEM (standard error of the mean).

## Results

### Cellular expression of M_1_-M_5_

**Figure 1:**
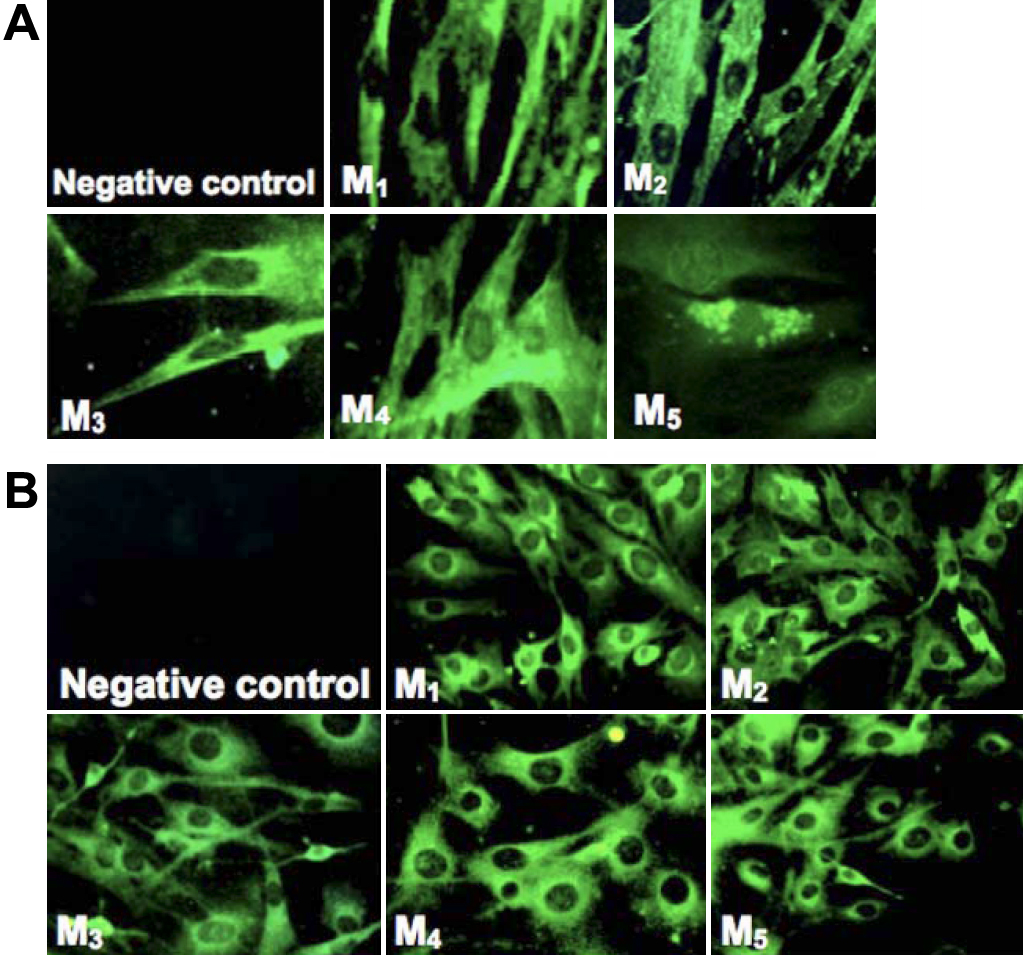
Immunohistochemistry of muscarinic receptor subtypes in early passages of cultured human and mouse scleral fibroblasts. **A **is the image of the early passage of cultured human fibroblasts, and **B **is the image of the early passage of mouse scleral fibroblasts. Subtype selective antibodies bound to cultured cells demonstrated the presence of the muscarinic receptors M_1_-M_5_ (shown in green). When primary antibodies were omitted, no binding was observed (negative control). The M_1_-M_5_ receptors were localized to the cell membrane as well as to the cytoplasm. Magnification, 200X. All experiments were performed in triplicate.

**Figure 2:**
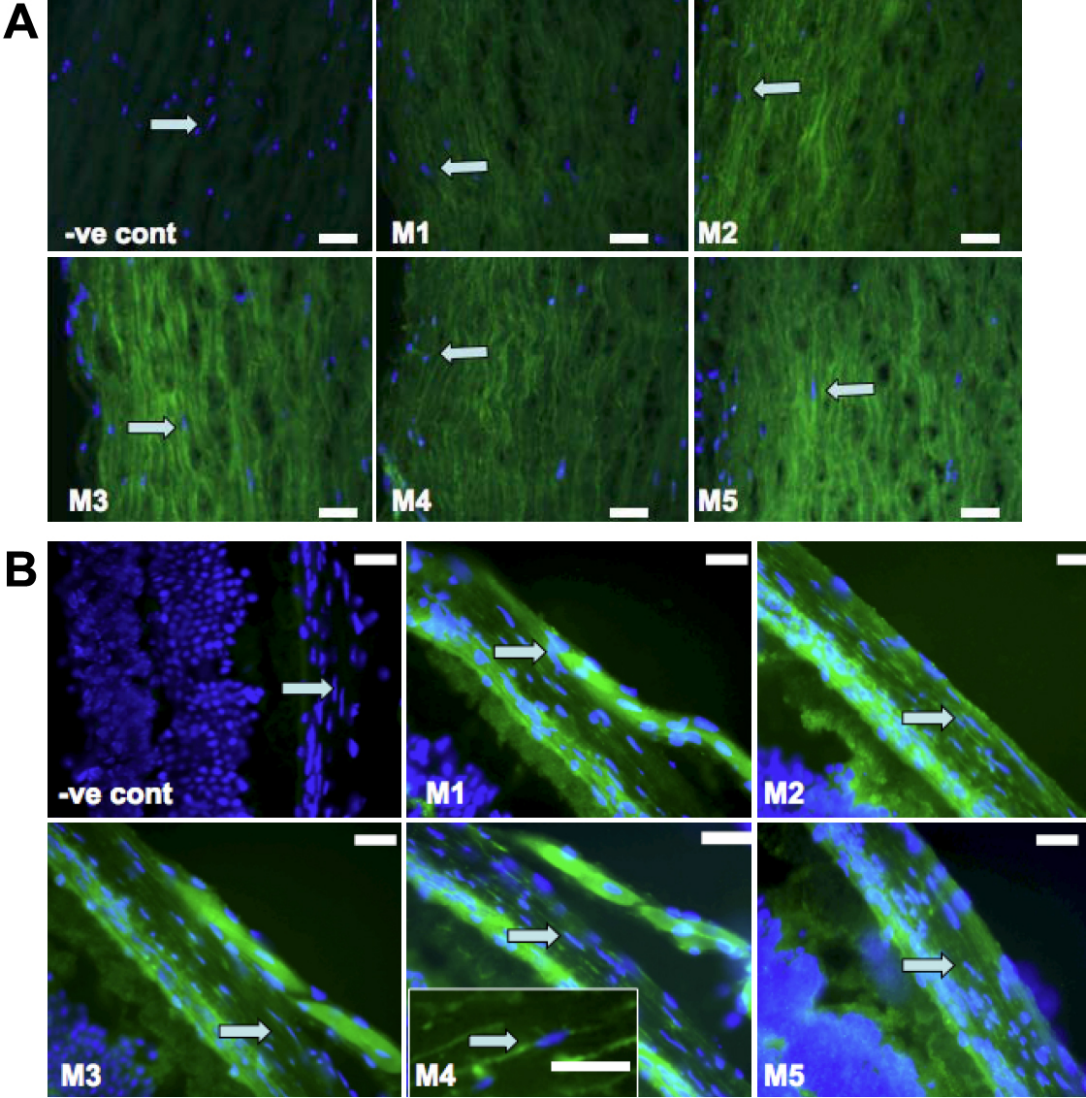
Immunohistochemistry of mAChR subtypes from human and Balb/c mouse. **A **shows the image of the human mAChR subtypes, and **B **illustrates the image of the Balb/c mouse mAChR subtypes. Subtype selective antibodies bound to scleral fibroblasts (arrowheads) demonstrates the presence of M_1_-M_5_ receptors (shown in green and nucleus shown in blue; stains with DAPI) at magnification 400X. When secondary FITC-labeled antibody was used without the primary antibody, no significant binding was observed (-ve cont means negative control). Arrow indicates scleral fibroblast. Scale bar=50 μm. Inset in **B**: enlarged image of fibroblast. All experiments were done in triplicate.

Immunohistochemical localization of muscarinic receptors M_1_-M_5_ was performed in passage 2-cultured human and mouse scleral fibroblasts (SFs) as well as scleral tissues. Positive immunostaining for all five mAChR subtypes was shown in the cultured human and mouse SFs (Figure 1A,B, respectively) as well as in human and mouse scleral tissues (Figure 2A,B, respectively). The M_1_-M_5_ receptors were localized to the cell membrane as well as to the cytoplasm. No immunostaining was observed in the negative controls. Thus, all five receptor types were represented in mouse and human scleral fibroblasts at passage 2 as well as in the mouse scleral tissue.

### Gene expression of muscarinic receptor subtypes

**Figure 3:**
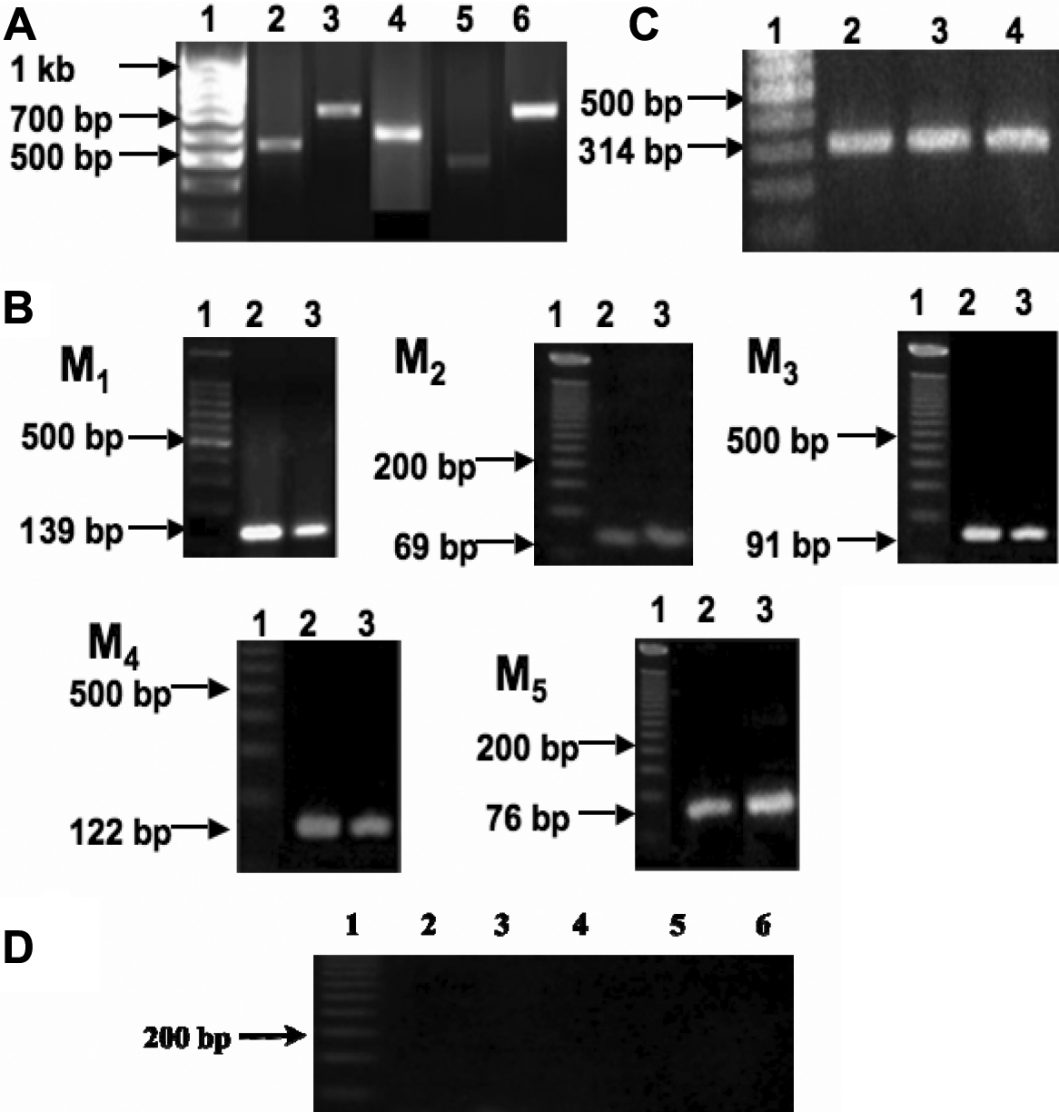
Gene expression of muscarinic receptor subtypes **A**: RT–PCR identified cDNA for *M_1_*-*M_5_* in human scleral fibroblasts. Lane 1: DNA ladder; lane 2: *M_1_*; lane 3: *M_2_*; lane 4: *M_3_*; lane 5: *M_4_*; lane 6: *M_5_*. PCR products of the expected sizes were as follows: *M_1_*, 538 bp; *M_2_*, 654 bp; *M_3_*, 560 bp; *M_4_*, 503 bp; and *M_5_*, 752 bp. The identity of the products was confirmed by sequencing. **B**: RT–PCR identified cDNA for *M_1_*-*M_5_* in mouse scleral fibroblasts. Lane 1: DNA ladder; lane 2: mouse brain as a positive control; lane 3: mouse sclera. PCR products of the expected sizes were as follows: *M_1_*, 139 bp; *M_2_*, 69 bp; *M_3_*, 91 bp; *M_4_*, 122 bp; and *M_5_*, 76 bp. **C**: *β-actin* as internal control, 314 bp for all cDNAs used. Lane 1: DNA ladder; lane 2: mouse brain; lane 3: human sclera; lane 4: mouse sclera. **D**: Water was used as a negative control. Lane 1: DNA ladder; lane 2: *M_1_*; lane 3: *M_2_*; lane 4: *M_3_*; lane 5: *M_4_*; lane 6: *M_5_*. Identity of the products was confirmed by sequencing (see Methods for detailed information).

RNA (1 µg) sample from each collected tissue (n=10 sclera) was separated on a 1.2% agarose gel to determine the RNA purity before further analysis. By conventional PCR, expression of all five mAChRs was detected in human and mouse sclera (Figure 3A,B, respectively). The identity of each PCR product was further confirmed by sequencing. Mouse primer sequences showed a high sequence identity to the NCBI Blast mAChR subtypes of mouse sequences (M_1_ 99%, M_2_ 100%, M_3_ 100%, M_4_ 100%, M_5_ 100%). As none of the five mAChR genes has introns, the PCR product from genomic DNA would yield the same-sized amplicons. To rule out possible contamination by genomic DNA, we chose a pair of *β-actin* primers at two adjacent exons that spanned a 206 bp intron on cDNA derived from *β-actin* mRNA. The identification of a single reverse transcription polymerase chain reaction (RT–PCR) product of predicted size for *β-actin* mRNA rules out contamination by genomic DNA. The *β-actin* primers were designed to span a 206 bp intron, finding a single band of 314 bp verifies that genomic DNA was not present.

**Figure 4:**
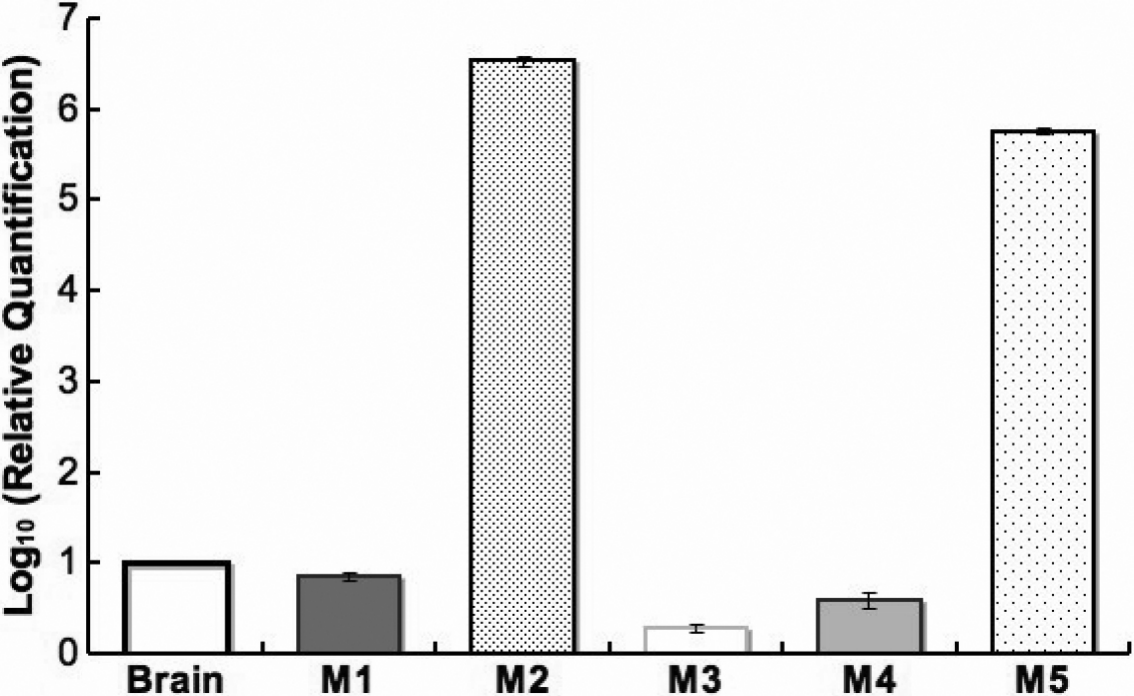
Muscarinic receptor sub-types transcript levels in mouse sclera. The bar graph compares the relative gene expression of mouse scleral muscarinic receptor subtypes to the corresponding mouse brain mRNA level (range) after normalization with 18S rRNA internal standard. The mRNA level of *M_1_*, *M_3_*, and *M_4_* in mouse sclera was less than in the mouse brain. However, the mRNA level of *M_2_* and *M_5_* was abundant in the mouse sclera.

Quantitative real-time PCR was used to compare the relative abundance of the transcript of each mAChR in the sclera. These levels were also compared to *M_1_*-*M_5_* gene expression in the mouse brain cerebellum. Normalized expression levels of *M_1_*, *M_3_*, and *M_4_* were lower in the sclera when compared to the levels for the mouse cerebellum (p<0.001). However, the levels of *M_2_* and *M_5_* were greater in the sclera when compared to the mouse cerebellum (p<0.05, n=10 sclera from five mice; Figure 4).

### Western blot analysis

**Figure 5:**
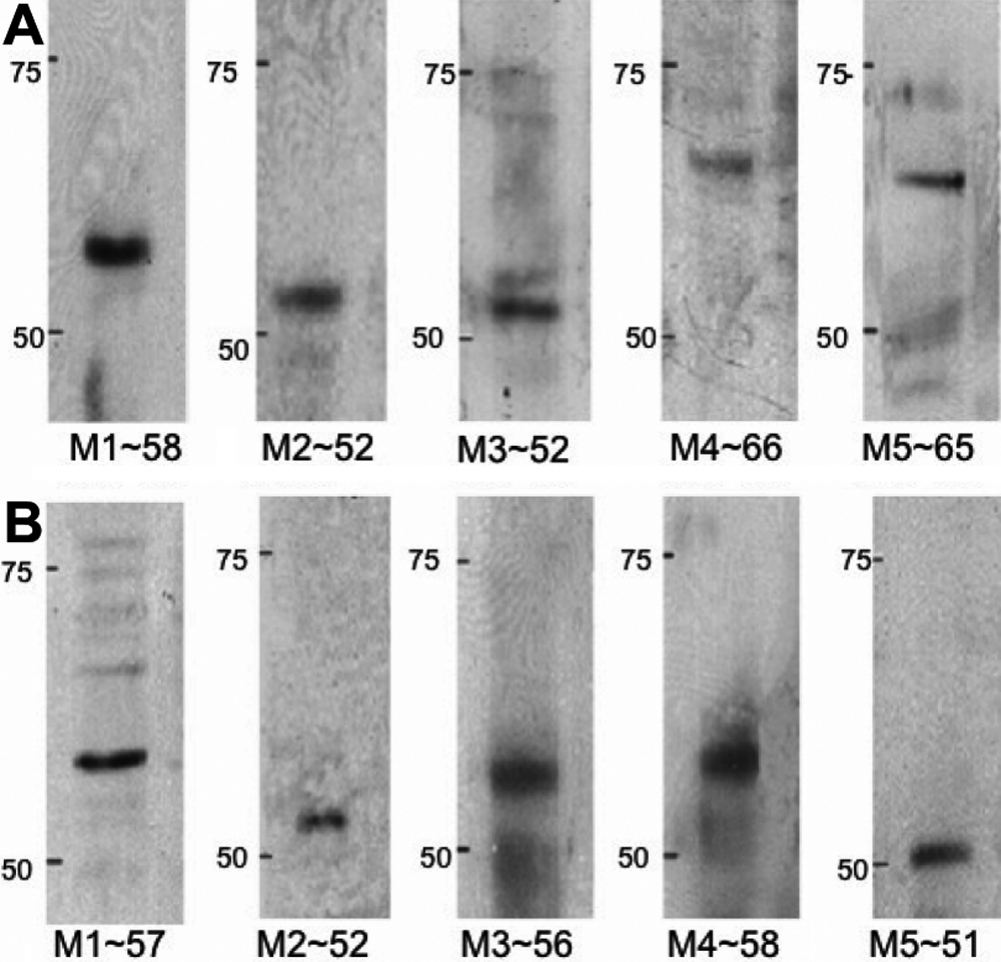
Muscarinic receptor protein expression. **A**: Immunoreactive bands corresponding to each muscarinic receptor subtype in mouse scleral fibroblasts and their estimated molecular weights are shown: ~58 kDa (M_1_), ~52 kDa (M_2_), ~52 kDa (M_3_), ~66 kDa (M_4_), and ~65 kDa (M_5_). Blots are representative data from at least three independent experiments. **B**: Immunoreactive bands corresponding to each muscarinic receptor subtypes in human scleral fibroblasts and their estimated molecular weights are shown: ~57 kDa (M_1_), ~52 kDa (M_2_), ~56 kDa (M_3_), ~58 kDa (M_4_), and ~51 kDa (M_5_). Blots are representative data from at least three independent experiments. Molecular standards (50-75 kDa) were run on the same blot parallel with muscarinic receptor proteins. The positions are shown in the left hand side.

Western blot analysis using SFs at passage 3 was performed using human and mouse cells to determine levels of protein expression of each muscarinic receptor subtype. Figure 5 shows that major bands representing M_1_ (58 kDa), M_2_ (52 kDa), M_3_ (52 kDa), M_4_ (66 kDa), and M_5_ (65 kDa) were detected. All five mAChR proteins were expressed in both mouse (Figure 5A) and human (Figure 5B) SFs.

### Muscarinic antagonists inhibited DNA synthesis

**Figure 6:**
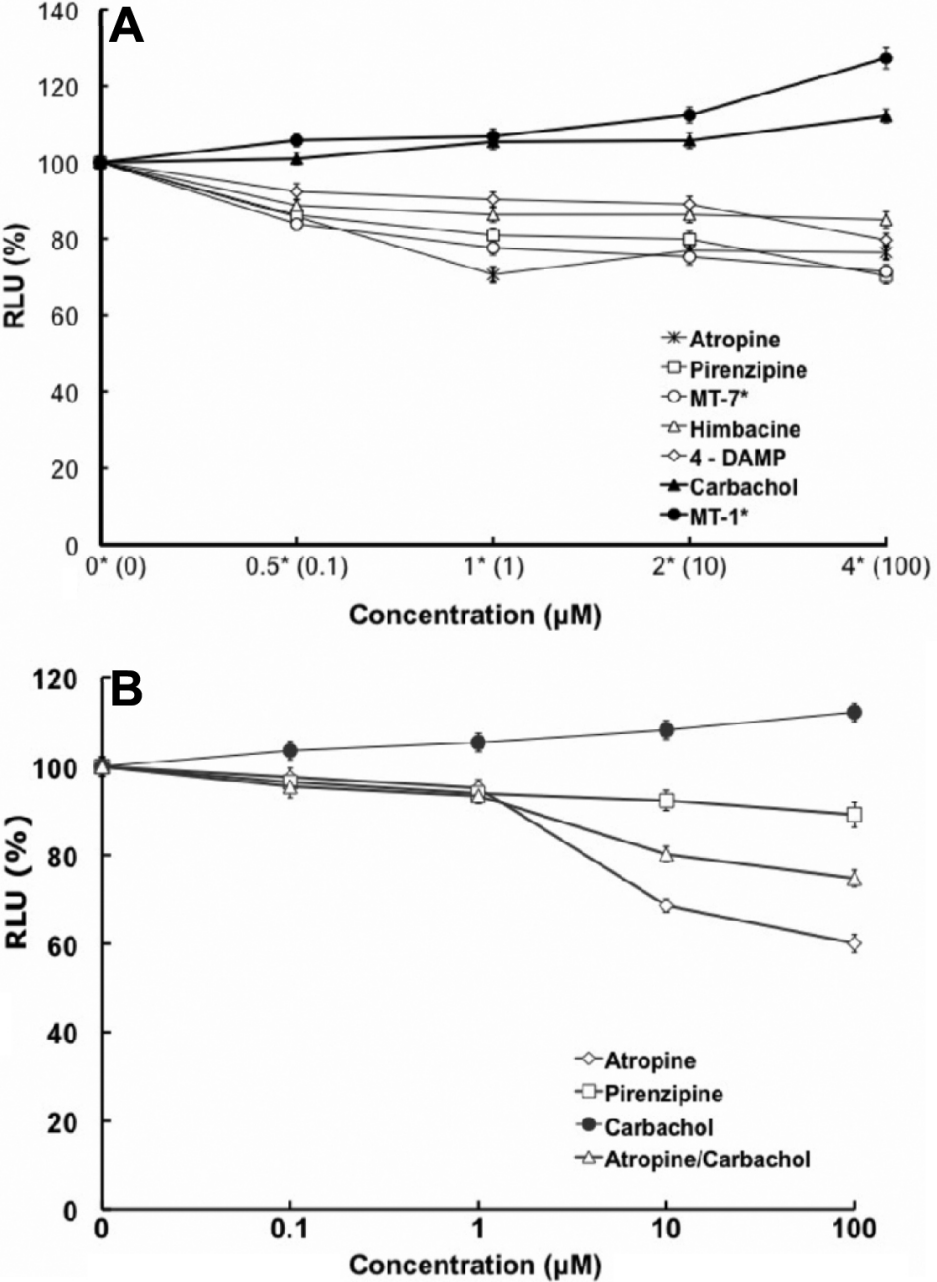
Muscarinic agents and mouse scleral fibroblast cell proliferation. **A**: The effect of muscarinic agents on mouse scleral fibroblast cell proliferation is illustrated on the graph. SFs were incubated with atropine, pirenzepine, carbachol, himbacine, and 4-DAMP at 0.1–100 μM and with muscarinic toxin-7 (MT-7), muscarinic toxin-1 (MT-1) at 0.1, 1, 2, 4 μM all for 24 h, and BrdU incorporation was measured by ELISA. Antagonists significantly inhibited DNA synthesis in a dose-dependent manner (p<0.05, ANOVA, n=4). In contrast, muscarinic receptor agonists, carbachol and MT-1, increased cell proliferation in a dose-dependent manner (p<0.05, ANOVA, n=4). Data are represented as mean±SEM. The asterisk indicates p<0.05 versus control (Post Hoc Analysis; Tukey Honest Significant Difference). **B**: Effects of muscarinic agents on cell proliferation of human scleral fibroblasts are shown. Scleral fibroblasts were incubated with atropine, pirenzepine, carbachol, and atropine/carbachol at 0.1–100 μM for 24 h, and BrdU incorporation was measured by ELISA. Antagonists significantly inhibited DNA synthesis in a dose-dependent manner (p<0.05, ANOVA, n=4). In contrast, muscarinic receptor agonist, carbachol, increased cell proliferation in a dose-dependent manner (p<0.05, ANOVA, n=4). Atropine was more effective at 10 and 100 μM than pirenzepine at all of the concentrations. Data are represented as mean±SEM. The asterisk indicates p<0.05 versus control (Post Hoc Analysis; Tukey Honest Significant Difference).

**Figure 7:**
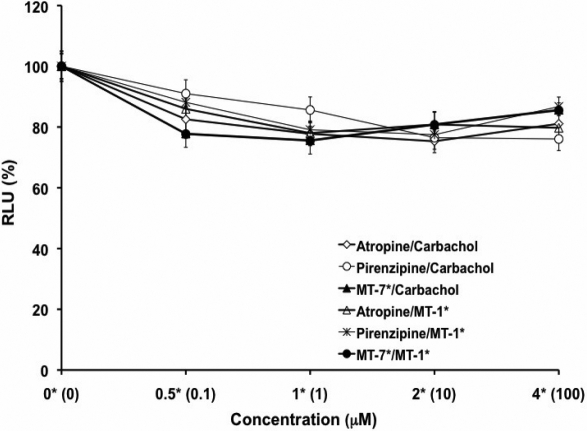
Effect of muscarinic antagonists in SF cell proliferation. Cell proliferation of mouse sclera fibroblasts were pre-incubated with atropine, pirenzepine at concentrations of 0.1, 1, 5, 10, and 100 μM, and MT-7 at 0.1, 1, 2, 4 μM for 3 h before they were incubated with equimolar carbachol at 0.1–100 μM and MT-1 at 0.1, 1, 2, 4 μM. BrdU incorporation was measured 24 h later by ELISA. Cells were treated together with antagonists and agonists. The antagonists blocked the agonist’s activation in a dose-dependent manner (p<0.05, ANOVA, n=4). Data represent mean±SEM. The asterisk indicates p<0.05 versus control (Post Hoc Analysis; Tukey Honest Significant Difference).

As muscarinic receptors are often linked to proliferation, we used this as an indicator of cell function of pharmacological control exerted through mAChRs. SFs were exposed to atropine, pirenzepine, or MT-7 for 24 h using a range of concentrations. By microscopic observation, cell morphology showed little differences between treatment groups and control groups, and viability as determined by the trypan blue exclusion assay showed that there was no toxicity after 24 h incubation of the cells with up to 100 μM of atropine (EC_50_=501.1 μm or 0.5011 mM) and pirenzepine (EC_50_=7040 μm or 7.040 mM) or 1 μM of MT-7. DNA synthesis, measured after 24 h using a BrdU ELISA assay, was inhibited after atropine, pirenzepine, and MT-7 exposure when compared with untreated control SFs (p<0.05, n=4; Figure 6A,B). In addition, when examining the competition between the agonists and antagonists, atropine, pirenzepine, and MT-7 blocked the effect of the action of carbachol (EC_50_=177.827 mM) and MT-1 (muscarinic agonist) when SFs were pre-treated (3 h) with an equimolar amount of antagonists (Figure 7). MT-7, which is selective for the M_1_, was almost as effective as atropine, a pan-muscarinic blocker, in decreasing BrdU uptake.

### Effect of muscarinic agents on MAPK activity

**Figure 8:**
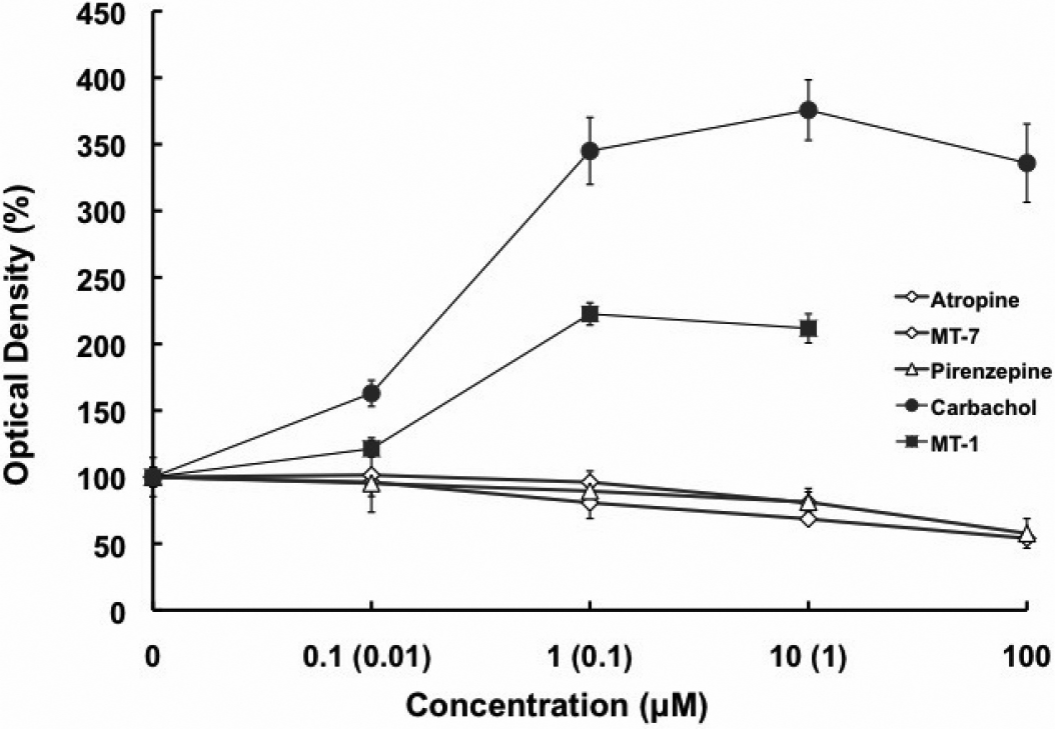
The activation of p42/44 MAPK in the muscarinic receptor-mediated responses in mouse SFs was analyzed via ELISA. The cells were exposed to atropine, pirenzepine, and carbachol at concentrations of 0.1, 1, 10, and 100 µM and to MT-7 and MT-1 at concentrations of 0.001, 0.01, 0.1, and 1 M. Phospho-p42/44 MAPK was activated in a dose-dependent manner (p<0.05, n=4). Data are represented as mean±SEM.

From the preceding data, it would be suggested that the MAPK pathways should be activated. We examined phosphorylation of p42/44 MAPK in mouse SFs following exposure to atropine, pirenzepine, and carbachol at concentrations of 0.1, 1, 10, and 100 µM and to MT-7 and MT-1 at concentrations of 0.001, 0.01, 0.1, and 1 µM using ELISA. Phospho-p42/44 MAPK was formed in a dose-dependent manner (Figure 8). Phospho-p42/44 MAPK decreased with the muscarinic antagonists and increased in response to the agonists (p<0.05, n=4).

### Immunoblot analysis

**Figure 9:**
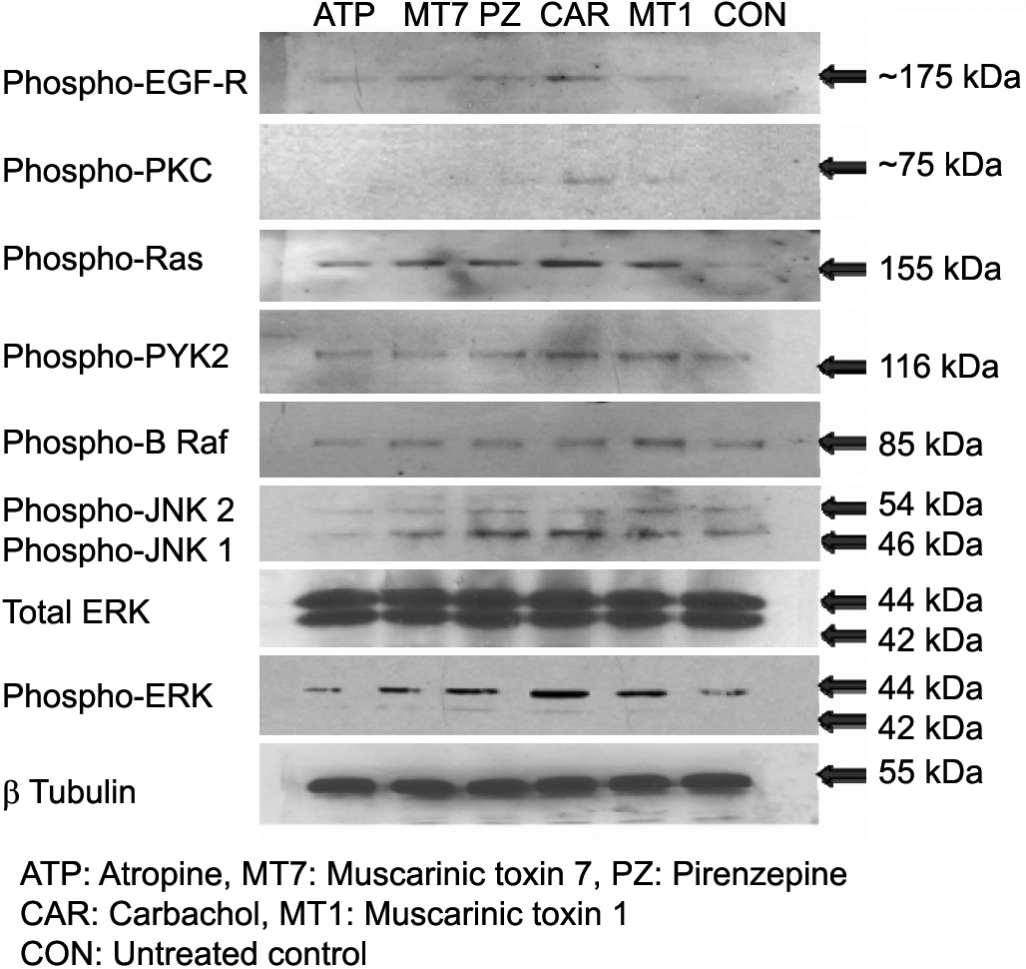
Effect of muscarinic agents on MAPK signaling proteins. Phospho-EGF-R, PKC, Pyk2, B-Raf, Ras, JNK1/2 and ERK1/2activity was detected by immunoblot in the cultured mouse SF cells after being treated with atropine, pirenzepine, and carbachol at a concentration of 50 µM and with MT-7 and MT-1 at a concentration of 0.5 µM all for 30 min. β-tubulin was used as a loading control for all the treatment. Antagonists increased their activation, and agonists reversed this.

The muscarinic receptors have been involved in trans-activation with tyrosine kinase receptors. We examined several specific points in the pathway to provide further information about the MAPK pathway. Phospho-EGF-R, -PKC, -Pyk2, -B-Raf, -Ras, -JNK1/2, and total/phospho-Erk1/2 were expressed at protein levels in mouse scleral fibroblasts following 30 min exposure to muscarinic agents, and β-tubulin was used as the loading control (Figure 9). The antagonists (atropine, pirenzepine, and MT-7) inhibited the formation of phospho-EGF-R, -PKC, -Pyk2, -B-Raf, -Ras, -JNK1/2, and -ERK1/2. In the absence of treatment, there was no effect on total Erk1/2, β-tubulin, or control. In contrast, in SFs that were treated with the agonists (carbachol and MT-1), the activation of phospho-EGF-R, -PKC, -Pyk2, -B-Raf, -Ras, -JNK1/2, and -ERK1/2  increased.

### Carbachol-induced Ras and ERK1/2 phosphorylation

We further demonstrated the carbachol-Induced Ras and ERK1/2 phosphorylation in mouse SFs to show the muscarinic agents also had parallel effects on the appropriate signal transduction pathways. As seen below, carbachol produced a time-and dose-dependent increase in Ras and MAPK/ERK phosphorylation in SFs.

**Figure 10:**
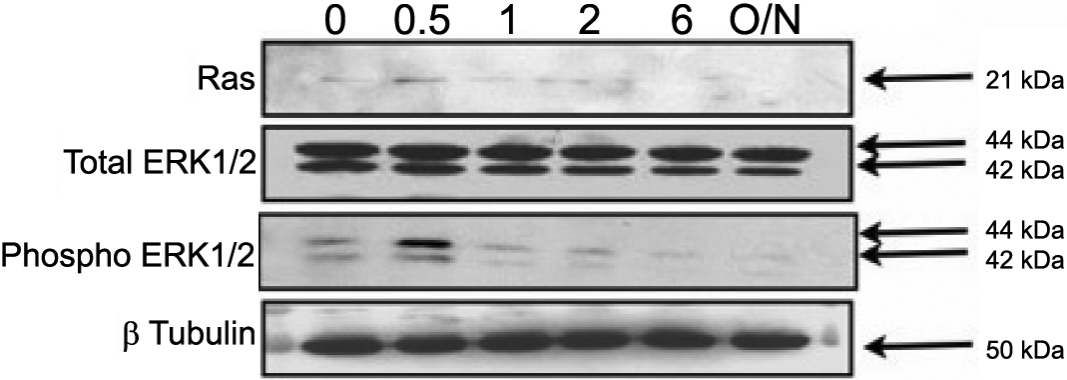
Activation of ERK1/2 and Ras by 50 mg/ml carbachol in cultured mouse scleral fibroblast cells. Cultured mouse SF cells were serum starved for 24 h in DMEM and then incubated with 50 mg/ml of carbachol for 0.5, 1, 2, and 6 h as well as overnight. Time-dependent change of phospho-p42/44 MAPK and Ras in the presence of 50 mg/ml of carbachol was detected by immunoblot analysis.

**Figure 11:**
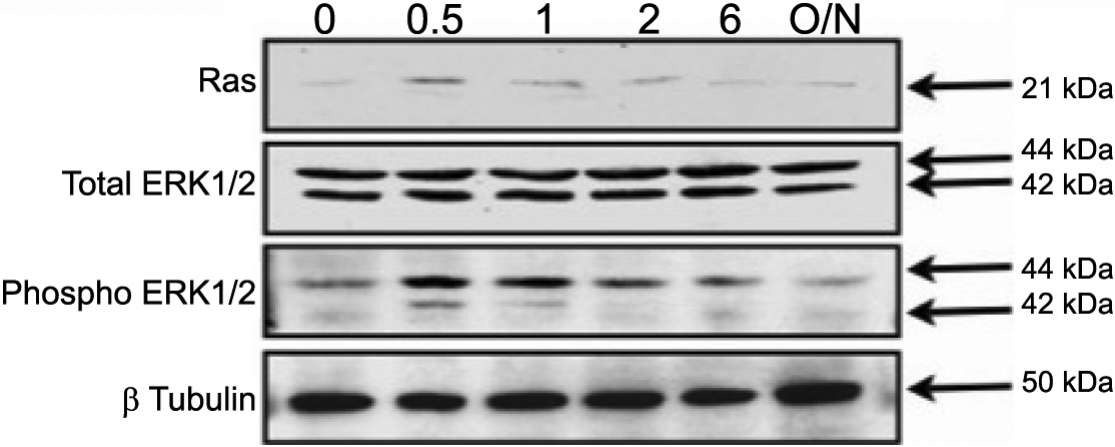
Activation of p42/44 MAPK and Ras proteins with FGF-2 treatment. Activation of p42/44 MAPK and Ras by 50 ng/ml of FGF-2 in cultured mouse scleral fibroblast cells was detected by immunoblot analysis. Cultured mouse SF cells were serum starved for 24 h in DMEM and then incubated with 50 ng/ml of FGF-2 for 0.5, 1, 2, and 6 h as well as overnight. Time-dependent change of p42/44 MAPK in the presence of 50 ng/ml of FGF-2 was detected.

We examined the activation of Ras and p42/44 MAPK in the muscarinic receptor-mediated responses in SF cells. Stimulation with 50 mg/ml of carbachol resulted in increased activity in Ras and p42/44 MAPK in a time-dependent manner (Figure 10). Ras and p42/44 MAPK activation was initially observed at 30 min with 50 mg/ml of carbachol stimulation (p<0.05, n=4). As a comparison to the activation of p42/44 kinase elicited by carbachol, we tested fibroblast growth factor (FGF-2) at 50 ng/ml, which was also found to activate Ras and p42/44 MAPK (Figure 11) with peak phosphorylation at 30 min (p<0.05, n=4). At 2 h, the level of Ras and phospho-p42/44 MAPK returned to basal levels (p<0.05, n=4). The p42/44 MAPK activation patterns for carbachol and FGF-2 were similar (Figure 10 and Figure 11).

### Carbachol stimulates EGF-R and TGF-β1 activity

**Figure 12:**
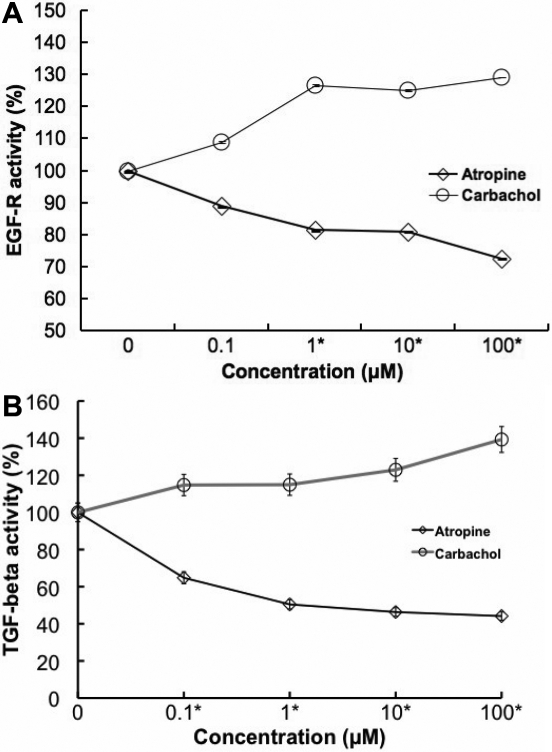
Muscarinic agents and Growth factor activity.** A**: EGF-R ELISA of mouse scleral fibroblast cell lysate after 24 h exposure to atropine and carbachol at 0, 0.1, 1, 10, and 100 µM found a dose-dependent change of EGF receptor in the presence of muscarinic agents. Carbachol stimulated EGF receptor activation in the cultured SFs. In contrast, the EGF receptor activation was inhibited by atropine in a dose-dependent manner (p<0.01, ANOVA, n=4). **B**: The results of TGF-β1 ELISA of mouse scleral fibroblast cell lysate after 24 h exposure to atropine and carbachol at 0, 0.1, 1, 10, and 100 µM is illustrated on the graph. Carbachol stimulated TGF-β1 activation in the cultured SFs. In contrast, the TGF-β1 activation was inhibited by atropine in a dose-dependent manner (p<0.01, ANOVA, n=4). Data are represented as mean±SEM.

Past study illustrated that scleral cells are directly responsible for DNA synthesis and extracellular matrix synthesis that produces axial elongation [33]. Muscarinic agents may act directly on sclera fibroblast through the G protein coupled muscarinic receptor to modulate postnatal eye development. We assume that the effect of muscarinic antagonists may also be mediated directly or indirectly by growth factors through receptor tyrosine kinases, which would then control sclera fibroblast activity. For these reasons, we focused on examining the activation of EGF receptor and TGF-β1 in the muscarinic receptor-mediated responses in SF cells. On stimulation with 0.1, 1, 10, and 100 µM concentrations of carbachol, EGF receptor, and TGF- β1 activities (Figure 12A,B, respectively) were increased in a dose-dependent manner. On stimulation with 0.1, 1, 10, and 100 µM concentrations of atropine, the EGF receptor and TGF-β1 activities were inhibited in a dose-dependent manner (p<0.05, n=4).

### Muscarinic antagonists modulates fibroblast growth factor-2 (FGF-2) activity

**Figure 13:**
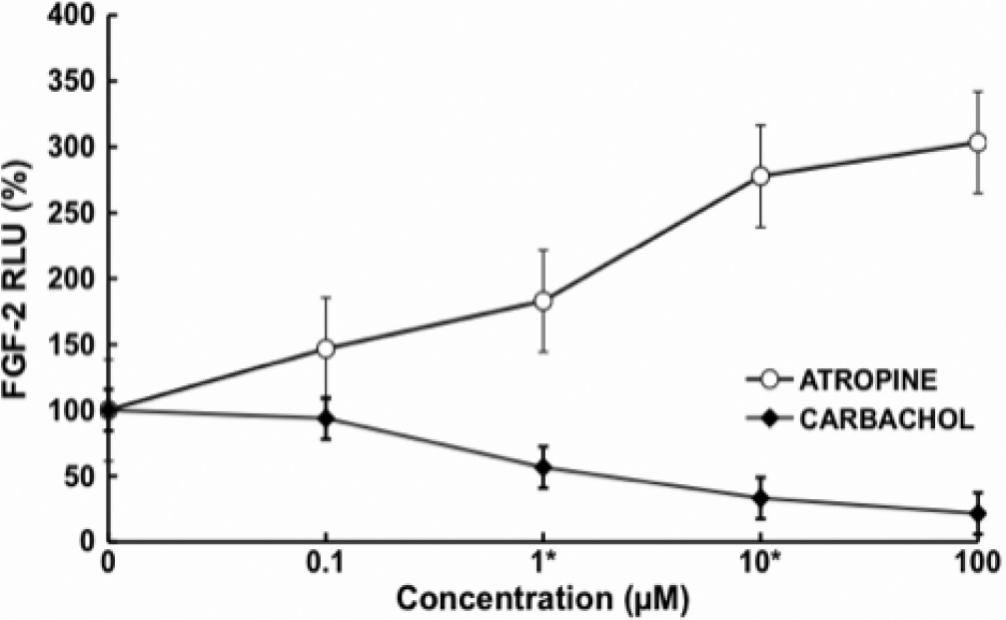
FGF-2 ELISA of mouse scleral fibroblast cell lysate after 24 h exposure to atropine and carbachol at different concentrations. Atropine increased FGF-2 activation in a dose-dependent manner. In contrast, carbachol decreased FGF-2 stimulation in a dose-dependent manner (p<0.05, n=4). Data are represented as mean±SEM.

In this experiment we showed that muscarinic agents modulate fibroblast growth factor expression in these cells. Atropine increased FGF-2 activation in a dose-dependent manner. In contrast, carbachol decreased FGF-2 stimulation in a dose-dependent manner (Figure 13A,B). Intraocular injection of FGF-2 can prevent axial elongation induced by form deprivation [34], suggesting that form deprivation might be associated with a reduction of FGF-2 or its receptors.

## Discussion

The fibroblast is the only cell type present in the collagenous scleral matrix, which is itself a product of these cells. Studies on scleral thinning and the involvement of the extracellular matrix [17] suggest that sclera elongation in experimental myopia involves more than just the weakening and stretching of sclera and requires biochemical and molecular changes for remodeling. The fundamental finding regarding these events is that atropine can retard myopia progression in humans [5,35]. As shown in this study, atropine affects both the muscarinic signaling as well as events mediated by growth factors. As the sclera fibroblast would be one of the first cell types to be contacted by a topical application of atropine as it moves into the eye, the mAChRs present in both human and mouse sclera makes it likely that the SF could be directly modulated by atropine.

Mouse SFs had higher levels of the M_2_ and M_5_ receptor when compared to other subtypes. It was reported in an earlier study that the M_2_ subtype is abundantly expressed in smooth muscle throughout the gastrointestinal tract [36] and in human lung fibroblasts [37]. The *M_5_* gene is abundantly expressed in ocular surface cells [31]. Studies [38] have reported that the message for *M_5_* predominated in cultured human lens cells with little or no expression of *M_1_* or *M_3_*. In contrast, in native lens cells, *M_1_* mRNA was in greatest abundance whereas in HLE-B3 cells [17] and the human brain [39], *M_3_* mRNA was predominant.

mAChRs are expressed in both neurons and glial cells of the central nervous system (CNS) and in various peripheral tissues [40-43]. CNS or neuronal mAChRs regulate a large number of important central functions including cognitive, behavioral, sensory, motor, and autonomic processes [44-46]. Studies of the mAChR in non-neural cells show that they are involved in the regulation of cellular proliferation in eye growth [47,48], tear fluid production [49], and lens cell signaling [50]. mAChRs were also shown to be involved in the modulation of keratinocyte proliferation as well as migration, cell-differentiation, and cell-to-cell contact in the skin [51]. Carbachol is able to stimulate proliferation in corneal epithelial cells [31] and human lung fibroblasts [37]. In the present study, a significant increase in BrdU incorporation was found with 50 mg/ml of carbachol in SF. The effect of carbachol was inhibited by pre-incubating SF cells with atropine, which is consistent with previous studies using human conjunctival cells [31]. We further demonstrated that M_1_, M_2_, and M_3_ subtype-selective antagonists alone blocked SF cell proliferation. However, the extent of inhibition of SF activity with the selective antagonists is different as compared with atropine. M_1_ subtype-selective antagonists, pirenzepine and muscarinic toxin MT-7, at concentration of 1 µM blocked SF cell proliferation by 81% and 78%, respectively, compared to 71% by atropine at 1 µM. In contrast, the M_2_/M_4_ antagonist (himbacine-86%) and the M_3_ subtype-selective antagonist (4-DAMP-90%) effect was greater than atropine. Our results show that the pan muscarinic antagonist of atropine was less effective in the inhibition of SF cell proliferation at a lower concentration. More specific subtype-selective antagonists are needed to study the molecular function of each subtype further. Children’s studies with 2% pirenzepine [6] showed that 2% pirenzepine was not as effective as 1% atropine [35] against myopia progression, which shows that the M_1_ receptor is not as critical in myopia as are some of the other types.

Epidermal growth factor (EGF) and transforming growth factor-β1 (TGF-β1) have a vital role in cell repair, stimulation of cell proliferation, cellular adhesion regulation, differentiation, hematopoiesis, apoptosis, tumorogenesis, migration, and extracellular matrix (ECM) production [52]. EGF functions via the epidermal growth factor receptor (EFG-R), a member of the ErbB family of receptor tyrosine kinases [53]. Tyrosine phosphorylation of EGF-R leads to the activation of the ERK/MAPK pathways [54]. EGF-R functions to transmit intracellular signals leading to regulation of cell growth. Alterations in the regulation of EGF-R function and overexpression of receptor or ligand results in cell proliferation. Anti-myopic effects of atropine may be mediated directly by muscarinic receptor or indirectly through growth factors such as FGF-2 and TGF-β, which then control SF cell proliferation. Growth factors modulate cell proliferation and composition of the extracellular matrix.

Carbachol-stimulated conjunctival epithelial cell proliferation correlates with the activation of p42/44 MAPK [27]. Our study showed that 50 mg/ml of carbachol activated p42/44 MAPK with kinetics similar to EGF-R. This was similar to the earlier study reported in corneal epithelial cells [31]. Carbachol-stimulated activation of p42/44 MAPK has been observed in goblet cells through increased intracellular Ca^2+^ concentration or transactivation of EGF pathways via phosphorylation of Pyk2 and Src [55,56]. Carbachol-induced contractions in the urinary bladder are reported to be mediated by G-protein coupled muscarinic M_3_ receptors [57]. M_3_ muscarinic receptors also mediate contraction in the guinea pig taenia cecum [58], and regulation of G protein levels could be playing a role in controlling muscarinic receptor activity in vivo [59].

**Figure 14:**
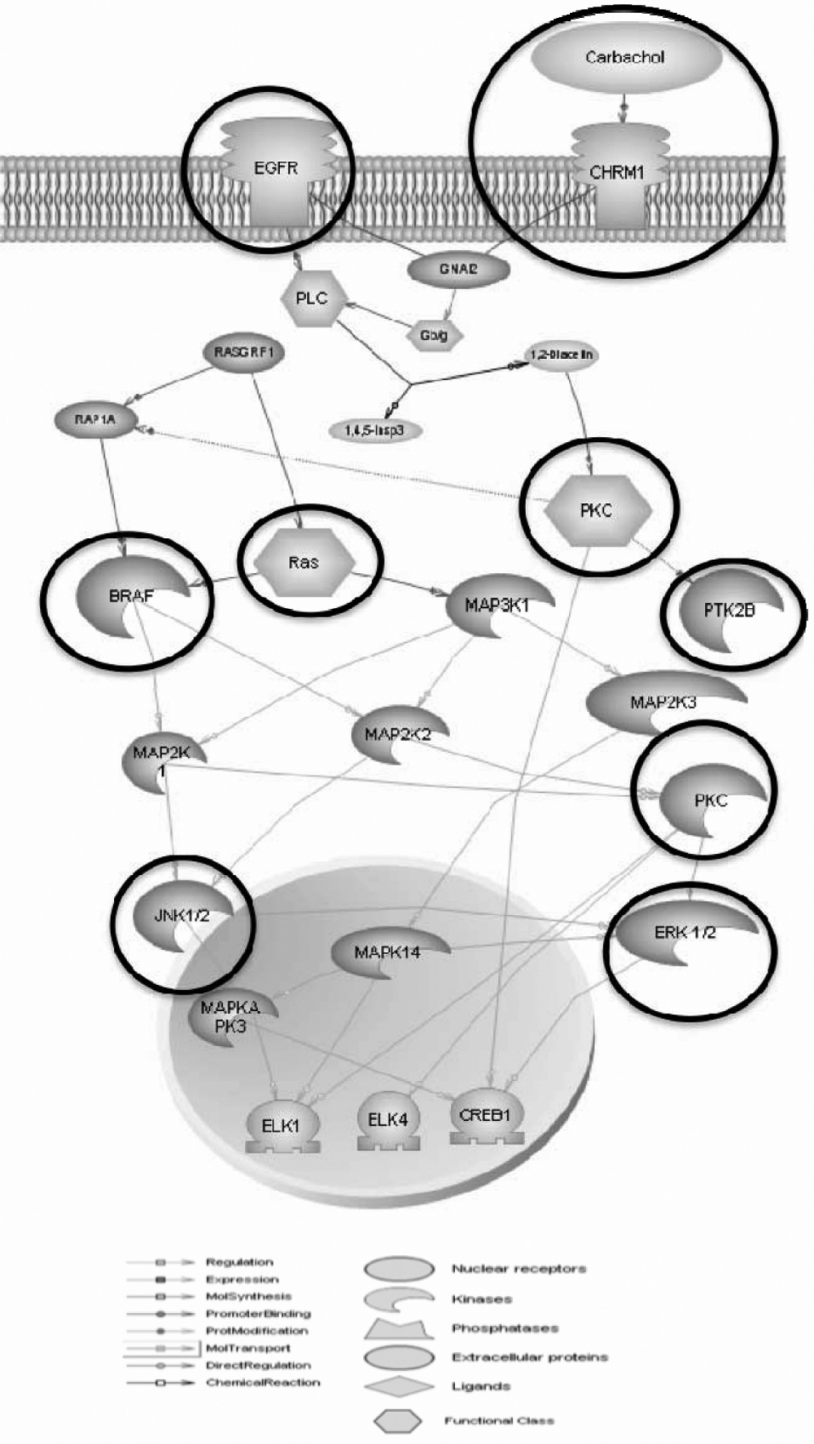
Muscarinic receptor-coupled signal transduction pathways mediating MAPK activity and proliferation in scleral fibroblasts. Both MAPK activity and cell proliferation are strongly activated by cholinergic (ACh) stimulation of mAChRs.

Selected entities from our studies were analyzed with interacting muscarinic pathways using pathway studio 6.0 (Ariadne Genomics, Rockville, MD; Figure 14) to explore the underlying intracellular signaling pathways involved in SFs treated with muscarinic agents. EGF-R is in the upstream, and all mAChRs are mediated via activation of the Ras-Raf-MAPK cascade. Both MAPK activity and cell proliferation are strongly activated by cholinergic (ACh) stimulation of mAChRs. Our data also support a role for G-protein coupled receptor-signaling pathway, which could be important for the muscarinic receptor mediated scleral cell proliferation [60].

In summary, our study showed the quantitative distribution of the five mAChRs in mouse SFs for the first time and also confirmed the presence of all five mAChRs in human and mouse SFs. Activation of mAChRs by carbachol led to increased SF cell proliferation. The mitogenic effect of carbachol correlated with the activation of p42/44 MAPK. The SF treated together with antagonists and agonists, the muscarinic antagonists blocked the agonist’s activation in a dose-dependent manner. Muscarinic agents modulate fibroblast growth factor expression in these cells. Atropine increased FGF-2 activation in a dose-dependent manner. In contrast, carbachol decreased FGF-2 stimulation in a dose-dependent manner. *M_2_* and *M_5_* genes are abundantly expressed in SFs and should be considered in the future design and selection of muscarinic receptor agonists or antagonists for use in topical eye drops.

It is shown that mAChRs mediate proliferation of human and mouse SF, a mechanism possibly involved in scleral remodeling. Blockade of these receptors might contribute to long-term beneficial effects of an anti-cholinergic drug in myopia.
